# Kick Study in Swimming Using Computational Fluid Dynamics

**DOI:** 10.3390/jfmk11040392

**Published:** 2026-09-29

**Authors:** Yefan Emma Jia, Zbigniew J. Kabala

**Affiliations:** 1 Pioneer Academics, 101 Greenwood Ave #170, Jenkintown, PA 19046, USA; 2 Department of Civil & Environmental Engineering, Duke University, Durham, NC 27708, USA

**Keywords:** Computational Fluid Dynamics, prosthetic, swimming, ankle and leg movement, hydrodynamic design, propulsion

## Abstract

**Background and Objectives:** For individuals with limb absence, staying active through sports can provide both social and physical benefits, with many turning to swimming. To prevent muscle imbalances and injuries, it is crucial to have a prosthetic that matches the propulsion of the intact limb, reducing strain on the intact limb. This study analyzes different foot movements in the crawl stroke kick to determine the most efficient model for swimming. **Methods:** This study is carried out through COMSOL 6.2 using Computational Fluid Dynamics to calculate the distance each model travels in one second. A sine wave is developed to define the foot rotation and the calf movement. Different phase differences of the foot movement relative to the original model are also tested. **Results:** The distance between each amplitude of the foot movement increases as the amplitude gets larger. For example, the difference in distance traveled between amplitudes of 0.4 and 0.5 radians is 0.84 cm, compared to 0.489 cm between 0.1 and 0.2 radians. A phase difference of 7π/4 produced the greatest distance traveled within one second. This analysis determines how close the original ankle technique in the crawl stroke is to the most efficient and powerful way of swimming. **Conclusions:** Within this simplified model, a prosthetic foot with a flexible ankle produced greater hydrodynamic propulsion than a fixed foot. These findings suggest that an ankle designed to more closely match the motion of the sound limb may be a favorable feature for sport-specific swimming prosthetics, offering a plausible strategy, grounded in the broader musculoskeletal literature, for reducing the risk of overstraining injuries associated with movement asymmetry.

## 1. Introduction

For those with limb absence, staying active through sports can provide both social and physical benefits. Aerobic training can allow for more efficient movement and decrease perceived levels of fatigue while walking [1]. A form of aerobic training recommended is swimming, yet swimming without a prosthetic can lead to more strain on the intact limb, causing muscle imbalances and possible injuries [2]. Injuries include osteoarthritis of the knee and/or the hip joint of the intact limb [3]. Because of this, the use of a well-fitted prosthetic can decrease the chance of injury by allowing the user to use both the intact limb and prosthetic limb equally. On top of this, using a prosthetic intended for swimming can help preserve the life of an individual’s daily prosthetic, which may be susceptible to damage from chlorine and water.

Current swimming-specific prosthetics for below-knee amputees face two competing, largely unresolved limitations: one being a type of fin reflecting the movement of flippers, and another typically consisting of a prosthetic limb and a fixed foot to allow for walking to and from a pool. Although the fin shaped prosthetic can allow for greater swimming power compared to a prosthetic with a fixed foot, it does not allow users to easily switch from land to water, and often users will need the assistance of others in order to get to a swimming location [4]. Fixed-foot designs, by contrast, are more practical for land-to-water transitions but fail to match the propulsive power of the sound limb. If the power generated from the prosthetic does not accurately match the sound leg, it can lead to strain on the intact limb that will only be furthered if the user wishes to swim more regularly and competitively, resulting in asymmetric loading and an elevated risk of injury [5]. This study is motivated by the second of these limitations: even among fixed- or flexible-foot designs, no swim-specific prosthetic has been optimized, using a systematic and repeatable approach, to match the propulsive characteristics of the flutter kick performed by the sound limb.

To appreciate what a swimming prosthetic must replicate, even partially, it is worth recognizing what an extraordinary engineering structure the natural foot is. Comprising 26 bones, 33 joints, and over 100 muscles, tendons, and ligaments [6], the human foot is one of the most mechanically complex structures in the body, accounting for roughly one quarter of all bones in the human skeleton. The vertebrate limb pattern itself has been shown to embody principles of universal optimal design, with each structural element serving a specific mechanical purpose [7]. This intricate architecture is what enables the foot to bear body weight, absorb impact forces of up to 2.5 times body weight during running, maintain balance across uneven terrain, and generate the propulsive forces needed for walking and running. Indeed, the ankle-foot complex in particular has been described as a masterpiece of engineering, with its multi-joint coordination, tendon energy storage, and arch mechanics working in concert to achieve efficient locomotion [8].

**Figure 1:**
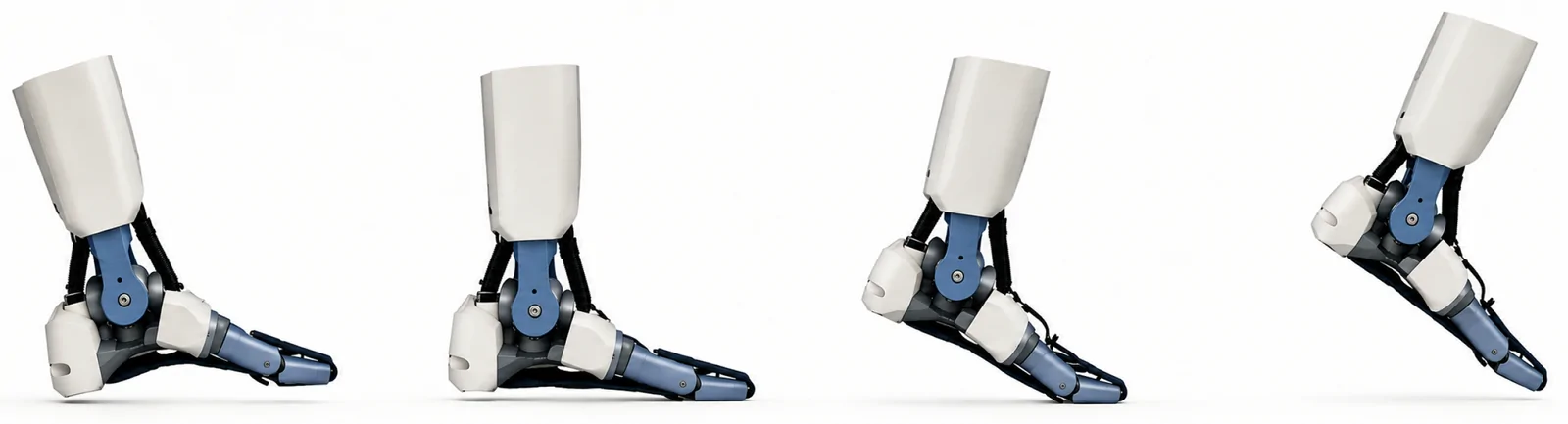
Bio-inspired foot prosthetic during the gait cycle, illustrating the multi-joint articulating design required for walking, running, and standing, inspired by [6]. Such full mechanical complexity is likely unnecessary for swimming, where propulsion demands are fundamentally different.

State-of-the-art bio-inspired prosthetic feet, such as the multi-articulating design shown in Figure 1, attempt to replicate this complexity through engineered joints, carbon-fiber leaf springs, and articulating ankle mechanisms, precisely because such complexity is indispensable for the demands of walking, running, and standing [6,8]. Robotic and bio-inspired designs have similarly sought to replicate multi-joint coordination for agile limb movement [9], including arched feet with individual toe joints and a plantar-fascia analog [10]. For swimming, however, the full mechanical complexity of the land-based foot is likely unnecessary. The foot does not need to bear body weight or absorb ground-reaction forces in water. Nevertheless, certain dynamic foot motions do appear essential for generating propulsion during the flutter kick. In particular, pronation, the inward rolling rotation of the foot about the long axis of the leg, and asynchronous movement between the foot and the calf are likely key contributors to propulsion, as demonstrated by studies of analogous appendage mechanics in biological swimmers [11].

Designs of a prosthetic with a flexible ankle that could be locked in either a plantar flexion (foot parallel with body) or a dorsi-flexed position (foot perpendicular with body) have been suggested, but some studies [12] have concluded with the preference of a prosthetic with a fixed foot over the previous design mentioned, partially due to the inconvenience of having to change the foot angle when entering water, although this could also be due to factors outside of the main design such as material choice and weight. For prosthetics with fixed feet that do not rotate at the ankle, the issue of matching the power of the sound limb arises, especially if users pursue a greater intensity of training in the pool. Prosthetic designs have aimed to mimic human swimming motion [13,14], but this can become difficult, acknowledging the greater lack of control users have on their movement, especially in environments such as in water. Ultimately, there is immense significance in imitating human ankle-foot biomechanics in prosthetic development [15]. While this study focuses on the up-and-down rotation of the foot during the flutter kick, it is worth acknowledging that lateral (side-to-side) rotation of the foot also likely contributes to propulsion in healthy swimmers, even if its effect is secondary. This study isolates the sagittal plane movement as the primary driver of propulsion in the flutter kick, but future work should consider whether incorporating lateral rotation into prosthetic design could yield additional propulsive benefits. It is important to note that various swimming competitions do not allow the use of prosthetics while swimming [16], such as the Paralympics, but prosthetic development can still be of use outside of competition.

### 1.1. Methods of Testing Prosthetics

Smaller scale testing comparing different prosthetics intended for swimming has been conducted, but few have conducted generalized prosthetic design comparisons. Most prosthetic testing has been conducted through having swimmers with lower limb absence do specific activities, including swimming one lap and treading, with and without different prosthetics [14,17]. After, the users would rate and compare the comfort, security, and more about each prosthetic. However, due to the difference between each participant’s strengths and techniques, as well as small sample sizes, it can be difficult to attain accurate and generalized data on prosthetic designs. Also, the process of creating a prosthetic that fits each participant can be costly and take up time. Researchers [18] have also found that throughout studies already conducted surrounding swimming prostheses, many used a participant pool of fewer than five, and it is likely that the same participation pool of 13 people was used across several empirical studies. This makes it difficult to reliably test new prosthetics, due to a lack of participants and variety within users.

Computational Fluid Dynamics relies on using fine mesh from a model to calculate velocity, drag forces, etc., through a numerical solver [19]. CFD allows for unwanted factors to be excluded, giving an advantage compared to testing with participants. This avoids errors, and can provide analysis on the fluid behavior around a subject. Its ability to simulate fluid–structure interactions is important for this paper, addressing how mechanical movement can impact the flow of water around it. CFD has been used in many cases prior [11,20,21], including analyzing the thrust of differing hand movements in cases of the crawl strokes [22], as well as calculating the drag of a male model that attained accurate results within 4% of experimentally determined values [23]. Similarly, CFD analyses of the passive gliding phase of swimming have examined hydrodynamic drag using both two- and three-dimensional simulations of a static swimmer body [24,25].

### 1.2. Goals and Rationale

This study uses CFD to model simplified movements and examine the impact of nonlinear foot kinematics on swimmer propulsion. The goal is to gather information that can be applied to improve prosthetic design, enhance a prosthetic user’s swim experience and training, and reduce injury. For our research, we utilize a 3D model to improve the accuracy of the analysis of foot and kick movements.

Although there are studies that design and develop swimming prosthetics for those with lower limb loss [12,13,14], few focus on the motion of the flutter kick in the crawl stroke. These prior studies have largely centered on pilot testing of complete prosthetic devices with human participants rather than on quantifying and optimizing the specific ankle kinematics that drive propulsion during the kick itself. To our knowledge, no existing study applies CFD to systematically vary and optimize the amplitude (amp.) and phase of ankle rotation during the flutter kick in isolation. This study addresses this gap by using Computational Fluid Dynamics (CFD) to examine the propulsion and technique of the ankle during the flutter kick. It quantifies this movement and compares it with similar movements found in existing prosthetic designs. The study analyzes the optimal ankle technique for maximizing body propulsion. By using CFD, this simulation avoids the variability in skill, technique, and exhaustion that can occur when testing with real people.

This will aid in prosthetic design by guiding the desired movement of the foot during the flutter kick, helping swimmers with lower leg loss avoid injury due to muscle imbalance and better match the movement of the intact limb. This is particularly important for those who train regularly and competitively, where technique is crucial and extended training increases the risk of injury with an ill-fitting prosthetic.

## 2. Materials and Methods

### 2.1. Theory

Computational Fluid Dynamics (CFD) involves mathematically predicting physical fluid flow by solving governing equations using computational power. The most common CFD tools are based on the Navier–Stokes (N-S) equations. CFD has become a widely applied tool for predicting real-world physics. These governing equations are based on the conservation laws of mass and momentum. For an incompressible fluid, they are as follows [26]:

(1) $$\rho g_{x}-\frac{\partial P}{\partial x}+\mu \left(\frac{\partial^{2}u}{\partial x^{2}}+\frac{\partial^{2}u}{\partial y^{2}}+\frac{\partial^{2}u}{\partial z^{2}}\right)=\rho \frac{Du}{Dt}$$

(2) $$\rho g_{y}-\frac{\partial P}{\partial y}+\mu \left(\frac{\partial^{2}v}{\partial x^{2}}+\frac{\partial^{2}v}{\partial y^{2}}+\frac{\partial^{2}v}{\partial z^{2}}\right)=\rho \frac{Dv}{Dt}$$

(3) $$\rho g_{z}-\frac{\partial P}{\partial z}+\mu \left(\frac{\partial^{2}w}{\partial x^{2}}+\frac{\partial^{2}w}{\partial y^{2}}+\frac{\partial^{2}w}{\partial z^{2}}\right)=\rho \frac{Dw}{Dt}$$

(4) ∇·V=0

where *x*, *y*, and *z* are Cartesian spatial coordinates; ρ is density; *t* is time; *g* is gravitational acceleration; μ is viscosity; and *P* is pressure; *V* = {u,v,w} is the velocity vector, whereas its *x*, *y*, and *z* components are *u*, *v*, and *w*, respectively. The Navier–Stokes equations describe the motion of fluid based on Newton’s 2nd Law of Motion, and they are defined in Equations (1)–(3) listed above. Equation (4), derived from the conservation of mass, is a form of the continuity equation.

We use CFD and conduct our testing through COMSOL Multiphysics, a finite element analyzer, solver, and simulation software package developed by COMSOL AB (Stockholm, Sweden), a company founded by Svante Littmarck and Farhad Saeidi in 1986 [27]. COMSOL Multiphysics is specifically designed for coupled phenomena and multiphysics simulations, providing a unified workflow for electrical, mechanical, fluid, acoustic, and chemical applications. The software facilitates conventional physics-based user interfaces and enables the solution of coupled systems of partial differential equations (PDEs), making it particularly well-suited for the fluid–structure interaction problems central to this study. Its versatility has been demonstrated across a broad range of peer-reviewed applications, including urban atmospheric flow modeling [28], heat transfer in nanofluids within engineered channel geometries [29], membrane distillation for water treatment [30], and evapotranspiration analysis in arid urban environments [31]. In addition, its use in fluid dynamics and pressure modeling has been validated against other established CFD platforms [32].

### 2.2. Video Analysis

To analyze and mathematically define the movement of the foot during the crawl stroke, we utilize a video [33] from the YouTube channel Effortless Swimming, which provides a clear side view of Olympian Daniel Smith swimming a 100-m freestyle. Smith competed on the Australian Olympic Team in the 2016 Summer Olympics in the men’s 4 × 200 m freestyle relay. He also won a silver medal in the men’s 4 × 100 m medley relay [34] at the 2016 World Short Course Championships. His Olympic-level competitive background suggests that his flutter-kick technique is likely efficient and well-refined, lending some support to using his kick as a representative reference motion despite the analysis being derived from a single swimmer. The clip used is a short segment that clearly captures a kicking motion. As the kinematic model presented here is derived from a single swimmer and a short video segment, it may not capture the full range of variability in flutter-kick technique across swimmers.

**Figure 2:**
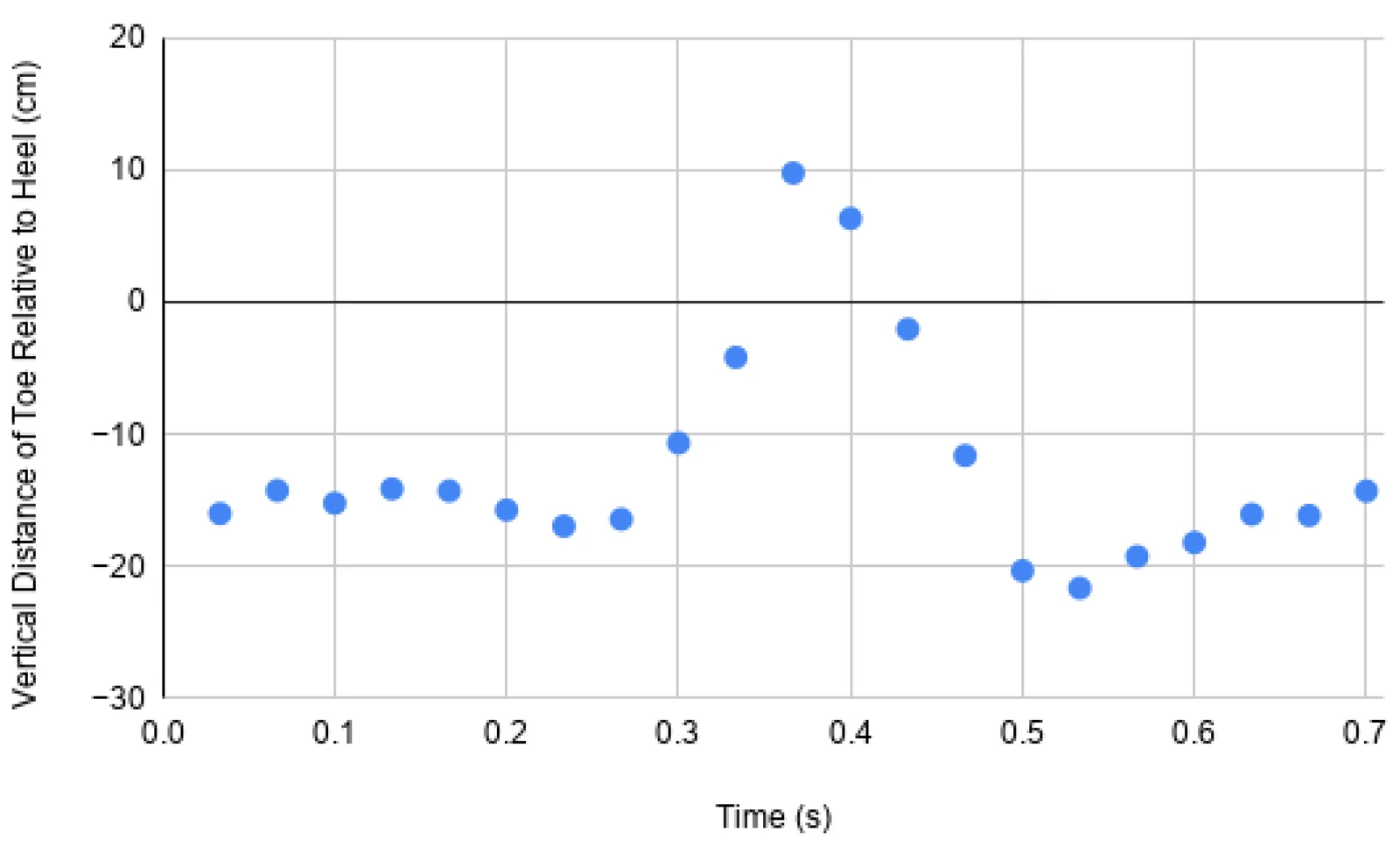
Vertical distance of toe relative to heel (cm) over time.

Using one segment of the video, the foot’s movement can be traced through the kicking motion, as the edges of the foot are well defined. To convert pixel coordinates in the video frame to physical units, each frame is calibrated using the average male calf length (40 cm) as a known reference length visible within the frame. This establishes a pixel-to-centimeter scale factor that corrects for frame-to-frame changes in camera distance or angle. The toe and heel positions are then manually digitized frame-by-frame, and the vertical distance between them is computed in physical units using this scale factor. The data collected is the vertical distance of the toe tip relative to the heel, which is plotted in Figure 2 to show how this movement changes over time. Additional segments can be used to confirm this foot movement.

**Figure 3:**
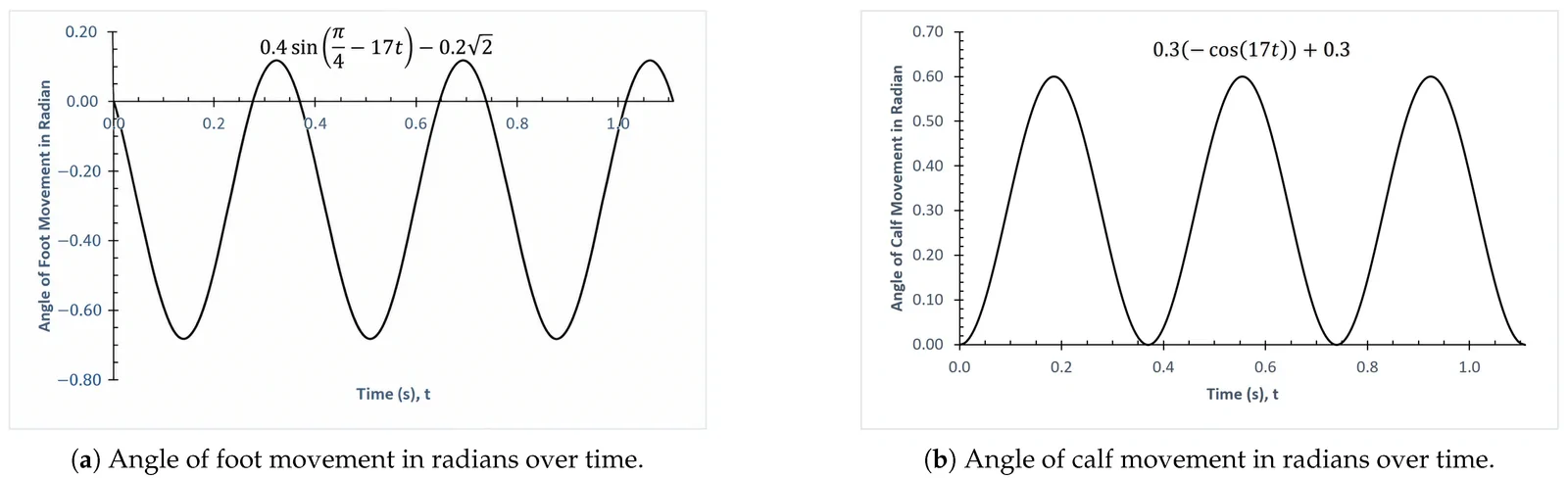
Angle of foot and calf movement in radians over time. (**a**) Foot movement. (**b**) Calf movement.

Based on the data above in Figure 2, the digitized vertical toe-to-heel distance is converted into an angular measure of foot rotation about the ankle, using the calf length as the effective lever arm. A sinusoidal function is then fit to this angular time series via nonlinear least-squares regression, with the amplitude, phase, and vertical-offset parameters optimized to minimize the residual between the fitted curve and the digitized data points. The angular frequency (17 rad/s in Figure 3a) corresponds to the swimmer’s observed kick cadence in the video. This yields the sine wave model shown in Figure 3a. The calf’s angular motion is digitized and fit in an analogous manner, yielding the cosine wave model obtained through the same digitization and fitting procedure, depicted in Figure 3b.

### 2.3. Model Configuration and Computational Set-Up

A 3D time-dependent study using a fluid–multibody interaction physics model is developed in COMSOL to simulate the movement of a swimmer’s foot and calf and analyze the interaction between the mechanism and the fluid. The study configurations include setting up the geometry, fluid interactions, and mesh.

#### 2.3.1. Geometry Set-Up

**Figure 4:**
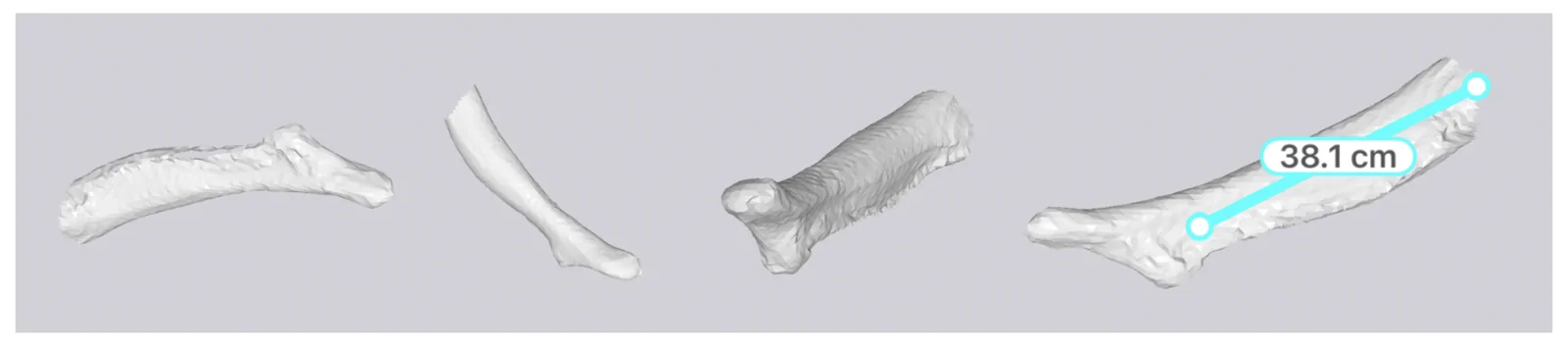
Three-dimensional scans of the calf and foot.

To create the geometry model for our simulation, 3D scans are taken using the 3D Scanner App v2.5, which employs LiDAR technology to capture 3D scans of the calf and foot from a human model, as shown in Figure 4. Under ideal conditions, these scans can be up to 95% accurate [35]. The calf, initially around 38.1 cm from the 3D scan, is then adjusted to 40 cm to fit the measurements from the video. The scanned foot has a length of 25 cm and, unlike the calf, is used unmodified in the simulation geometry, as it is the primary focus of this study. Because limb dimensions vary between individuals and directly affect the hydrodynamic scale of the simulation, Table 1 reports the calf and foot length used in every simulated trial, alongside the corresponding amplitude and phase-difference values; these dimensions are held constant across all trials reported in this study.

The scan is imported into COMSOL, where a cylinder-shaped calf, knee joint, and ankle joint are added to simplify the model while maintaining relative accuracy in length and size. This simplification is intentional because the primary aim of this study is to characterize the propulsive contribution of the foot and ankle motion specifically. Computational effort is concentrated on resolving the foot geometry, which is retained directly from the 3D scan, rather than on anatomically detailed modeling of the calf, thigh, or upper leg. Additionally, the scan is slightly edited to smooth out ridges and holes, particularly on the part of the calf facing upwards shown in Figure 4. The foot remains unadjusted as it is the main focus of this paper. Using Multibody Dynamics within COMSOL, hinge joints are defined to allow for the sine wave model movements. The calf and foot movements are defined separately, with the calf movement at the knee hinge joint and the foot movement at the ankle hinge joint. Constraints are applied at the knee and ankle to prevent unnecessary rotation as the calf and foot follow their defined rotation. Additionally, continuity is set at the connection surface between the calf and ankle joint to keep the multibody intact. This is a simplification of the present geometric model rather than a limitation of the underlying research question, which concerns the propulsive contribution of foot kinematics. Future work using a more anatomically continuous calf-ankle transition could help clarify how much of the local flow structure near this junction is an artifact of the simplified geometry versus a genuine feature of the flow around the ankle.

**Figure 5:**
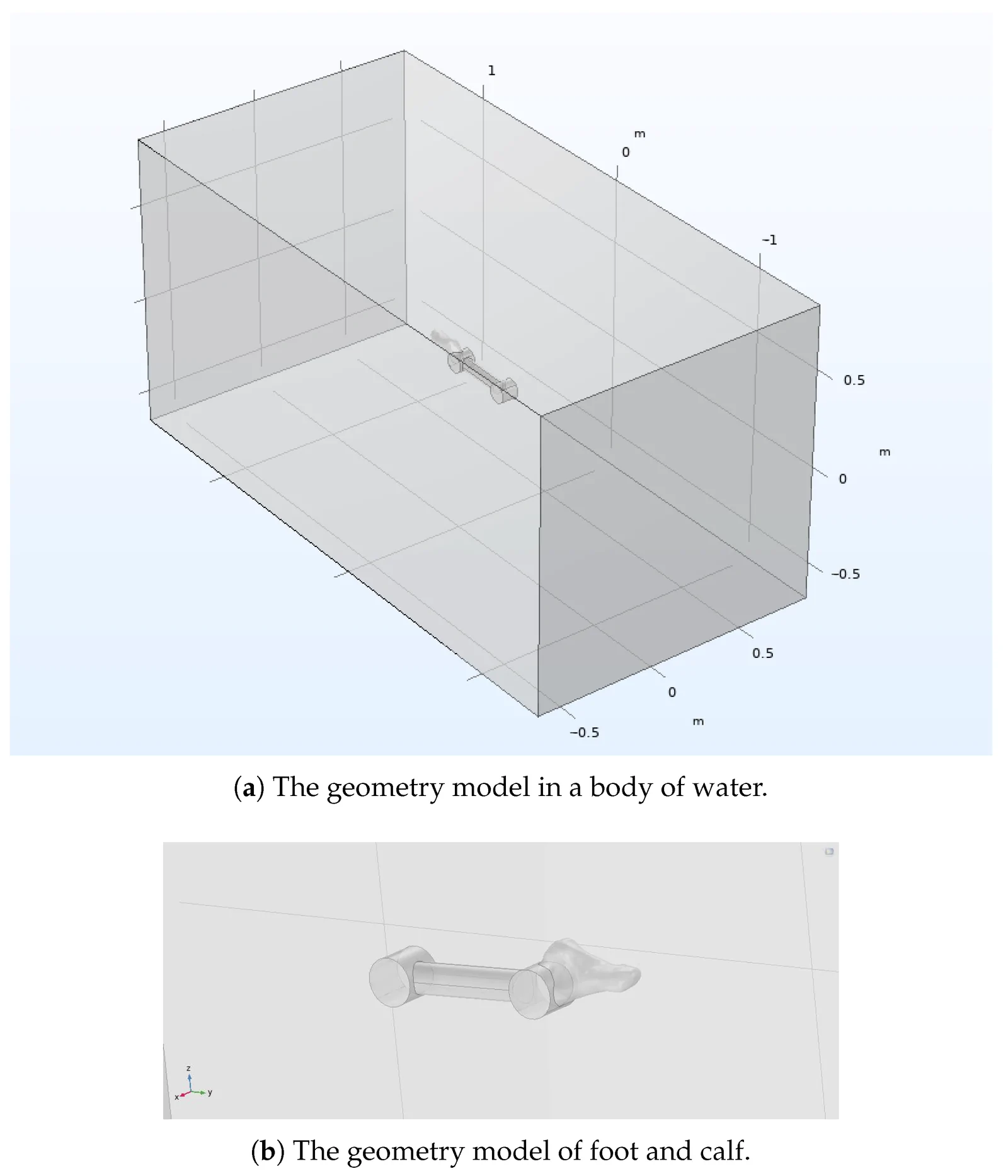
The geometry model. (**a**) Whole geometry. (**b**) Foot and calf geometry.

The geometry model is placed in a body of water with a depth and width of 1.5 m and a length of 3 m, consistent with the relative depth used in similar CFD setups [36]. The length is shorter because the model does not include the entire human body. The model is positioned in the center of this domain to better capture the fluid movement around it, as shown in Figure 5. A moving mesh is set up to allow for changes within the mesh as the model simulates a kick.

#### 2.3.2. Fluid Interactions

Fluid–Structure Interactions are incorporated into the setup to account for the relationship between the model and the fluid. This setup utilizes pairs created through Form Assembly during the geometry setup. It is based on the COMSOL-provided setup for a similar model [37] and has been modified to reflect the specific parts and movements involved. Laminar flow is used to create a water tunnel within the body, with the water velocity set to 0 m/s. The use of a laminar-flow model is justified by considering the Reynolds number of the flow generated by the kicking motion. We note that, as the characteristic length scale, we selected the calf width (0.1 m) rather than its length or the length of the foot. This choice is analogous to using the pipe diameter, rather than its length, as the characteristic length scale when calculating the Reynolds number for pipe flow. Using the width of the calf (0.1 m) as the characteristic length scale and the velocity of the kicking leg measured in the simulation (approximately 2.5 cm/s) as the characteristic velocity, together with the kinematic viscosity of water at 25 °C ($\nu \approx 0.893\times 10^{-6}$ m^2^/s), we calculate a Reynolds number of approximately Re ≈ 2800 for our simulation. This is consistent with precedent in the biological-swimming CFD literature: Fan et al. [38], based on the swimming velocity and limb dimensions of a swimming frog (*Xenopus laevis*), reported a Reynolds number of approximately 5000 for their self-propulsion CFD simulation, which they characterized as lying in the transitional region between laminar and turbulent flow. Notably, Fan et al. directly compared laminar and turbulent viscous models at this Reynolds number and found only a small discrepancy between the two; they therefore used the laminar model to simplify the simulation and reduce computing time.

Given that our own Reynolds number is two times smaller, and that a directly comparable biological-swimming CFD study explicitly tested both models and found the laminar assumption adequate in this same transitional regime, we believe laminar flow is a reasonable simplifying assumption for our simulations as well. We note, however, that our case differs from theirs in one respect: the oscillating, kicking motion of the foot and calf sheds vortices on each stroke, which may locally promote some transition toward turbulence that a body held in a fixed position under steady flow would not experience. We therefore flag the addition of a turbulence model (e.g., the *k*-ω SST model, as used by Samson et al. [39]) as a natural and valuable extension of this work for future studies, to directly test whether departures from the laminar assumption meaningfully affect the propulsion trends reported in Section 3.

The materials used are predefined within COMSOL, with the surrounding domain set to water and the model itself set to titanium beta-21S.

The fluid domain does not include an inlet or outlet, as the water is initially at rest and is set into motion entirely by the kicking motion of the model rather than by an imposed background flow. The two side walls, the bottom, and the top of the domain are all treated identically as slip walls, permitting tangential flow along these boundaries without a no-slip condition. In particular, the top boundary is treated the same way as the side and bottom walls, rather than as a free, deformable air-water interface; this is a simplification of the free-surface effects present near the water’s surface during an actual flutter kick, and is discussed further as a limitation in Section 5. The only no-slip boundary in the domain is the surface of the foot and calf model itself, where the Fluid–Structure Interaction pair couples the fluid stresses to the multibody dynamics of the model. The time-dependent study is solved over a range of 0 to 1 s using a fixed time step of 0.01 s. The coupled fluid–structure interaction problem is solved using a segregated solver approach with a direct linear solver at each time step. The moving mesh accommodating the deformation of the fluid domain around the kicking foot and calf uses hyperelastic mesh smoothing to control mesh distortion as the boundary moves through each time step.

#### 2.3.3. Mesh Set-Up

**Figure 6:**
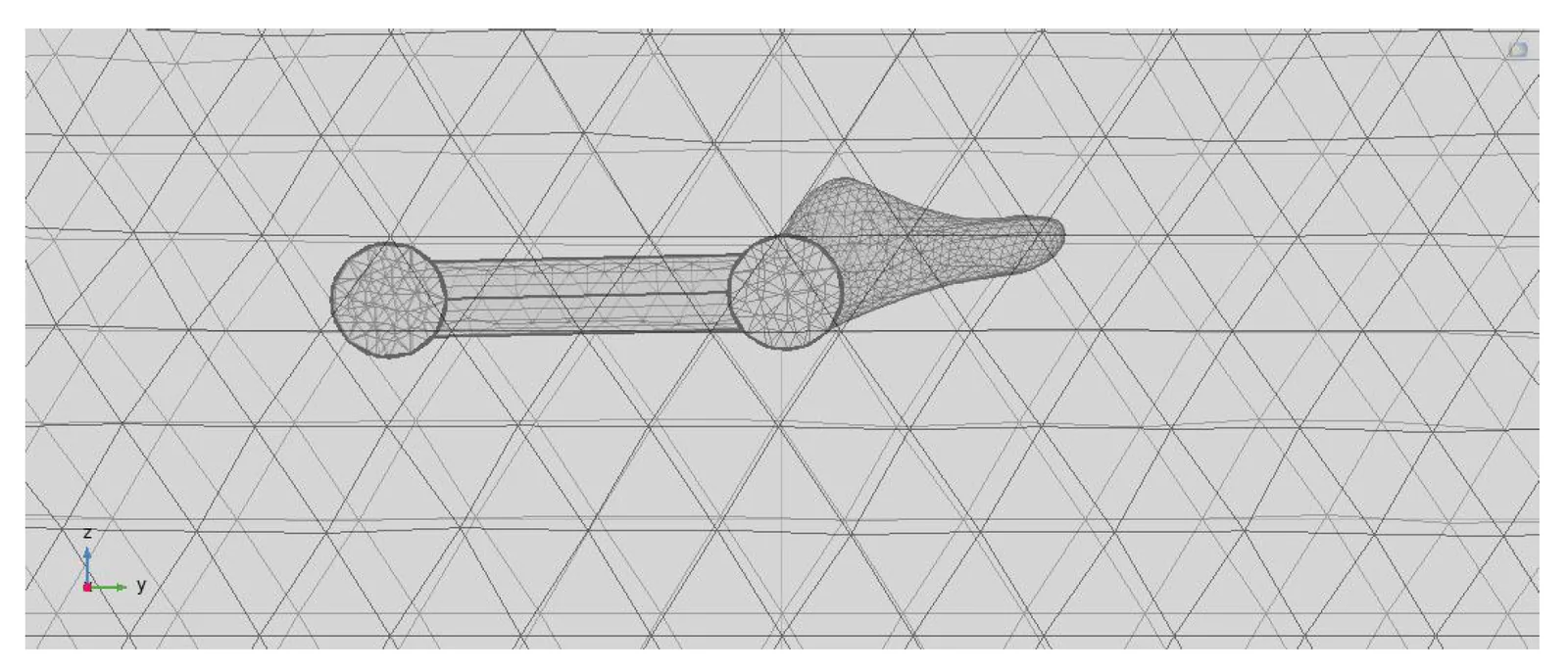
Mesh at t = 0.

The mesh element size for each boundary of the calf and foot model is set to finer, while the surrounding water is kept at a normal size. A Swept Mesh is used for the “calf” cylinder to prevent inverted mesh elements. Corner Refinement and Boundary Layers are applied to the water domain. The full geometry uses Free Tetrahedral, creating a finer mesh around the leg setup, with larger elements further away to optimize computation time, as shown in Figure 6.

### 2.4. Convergence Analysis

**Figure 7:**
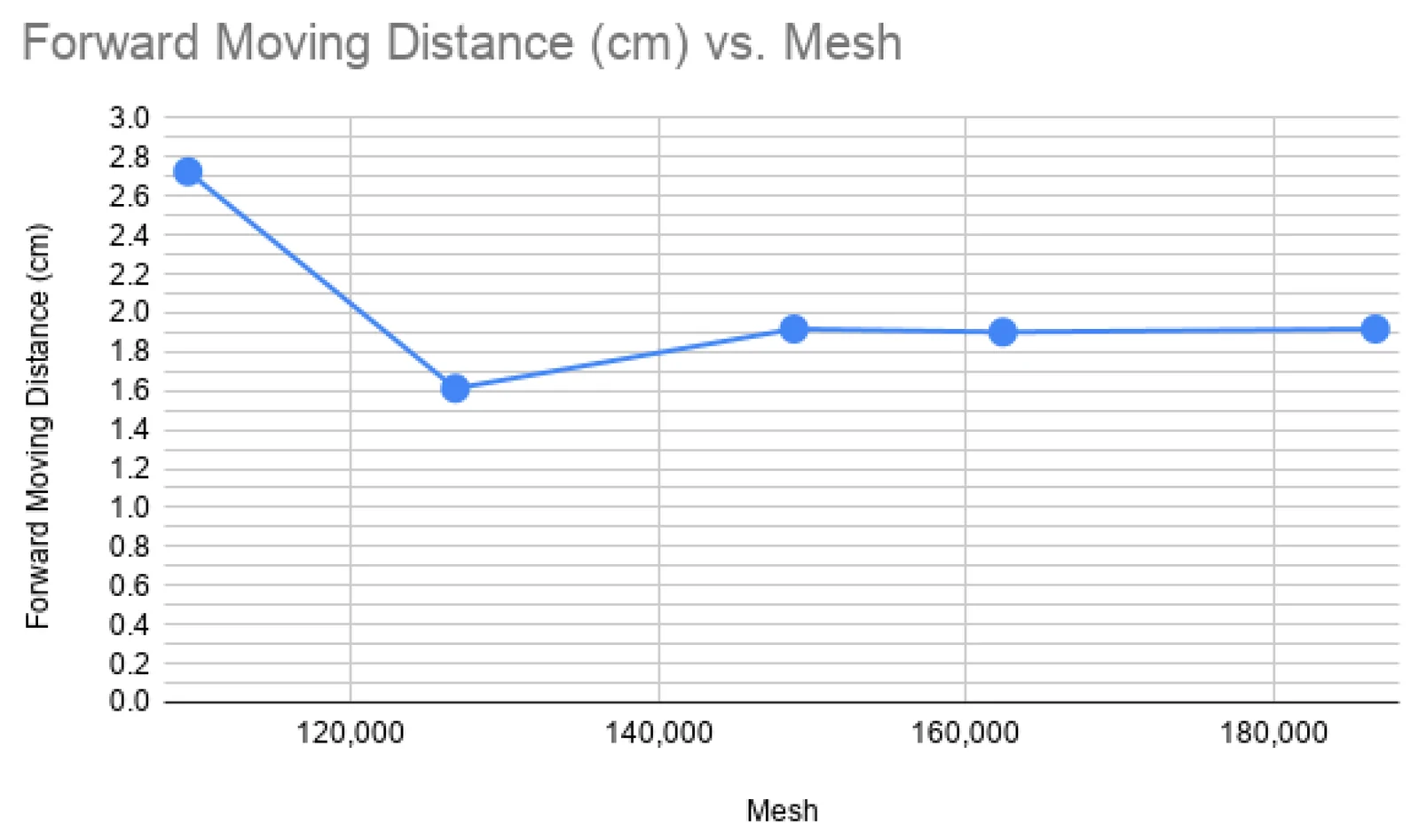
Forward moving distance (cm) versus mesh size.

To assess the numerical convergence of this study, a mesh convergence test is performed. The convergence is demonstrated in Figure 7 on the model defined above. Since results with more than 148,000 elements are independent and show less than a 1% change, the normal mesh resolution is used for the simulations to balance accuracy with computation time. This convergence test establishes the numerical independence of the simulation results from mesh resolution.

### 2.5. Previous Experimental Studies

No published CFD study modeling the ankle-foot flutter-kick motion in isolation, using a geometry and boundary-condition setup comparable to the one presented here, could be identified in the literature. In the absence of a directly comparable CFD benchmark, we instead compare the order of magnitude and qualitative flow behavior of our simulated results against independent experimental measurements of human flutter-kick propulsion. Pressure-sensor measurements of front-crawl flutter-kick thrust in competitive swimmers place peak kicking thrust on the order of 80–100 N [40], giving a physiologically relevant benchmark for the order of magnitude of the forces in our simulation. This is further supported by experimental measurements of lower-limb power output during flutter-kick swimming [41], which likewise indicate physiologically substantial force generation from the kick alone, independent of any CFD modeling assumptions. In addition, foot propulsion has been shown to reach a magnitude comparable to hand propulsion during front-crawl swimming [42], supporting the premise that ankle and foot motion are a meaningful, rather than secondary, contributor to propulsion. Furthermore, flutter-kick thrust is understood to arise from the formation and shedding of vortices around the foot [43,44], a mechanism that is qualitatively consistent with the flow structures we observe around the foot in our own simulations. Comparable vortex-based thrust generation has also been documented in CFD studies of dolphin swimming [45]. We note that these are physical and experimental measurements, not CFD studies of a matching geometry, so this comparison is intended to show that our simulated propulsion trends are physiologically plausible, rather than to serve as a direct quantitative validation.

## 3. Results

As previously mentioned, this simulation study aims to assess and quantify the impact of foot movement in the flutter kick with varying amplitudes and phase differences of the sine wave model defined in Figure 3a. For each trial, the calf movement remains consistent with the cosine wave model defined in Figure 3b. Displacement measurements are taken over a 1 s period, focusing on forward movement distance. The forward movement is measured by the change in the horizontal position of the front of the knee joint, as it does not rotate and can be tracked accurately. The findings are presented as follows:

### 3.1. Amplitude of Foot Movement

To determine the difference in propulsion between different foot movements, the amplitude of the sine wave model for the foot rotation around the ankle is adjusted, including 0 radians, 0.1 radians, 0.2 radians, 0.3 radians, 0.4 radians, and 0.5 radians. The sine wave equation is altered such that for each amplitude change, the starting point at time 0 s will be at position 0 to keep continuity. These changes are selected to reflect realistic human motion. Tests are not conducted with the foot remaining in a dorsi-flexed position, since the drag factors and much smaller horizontal surface area make it clearly the worst performing in propulsion.

**Figure 8:**
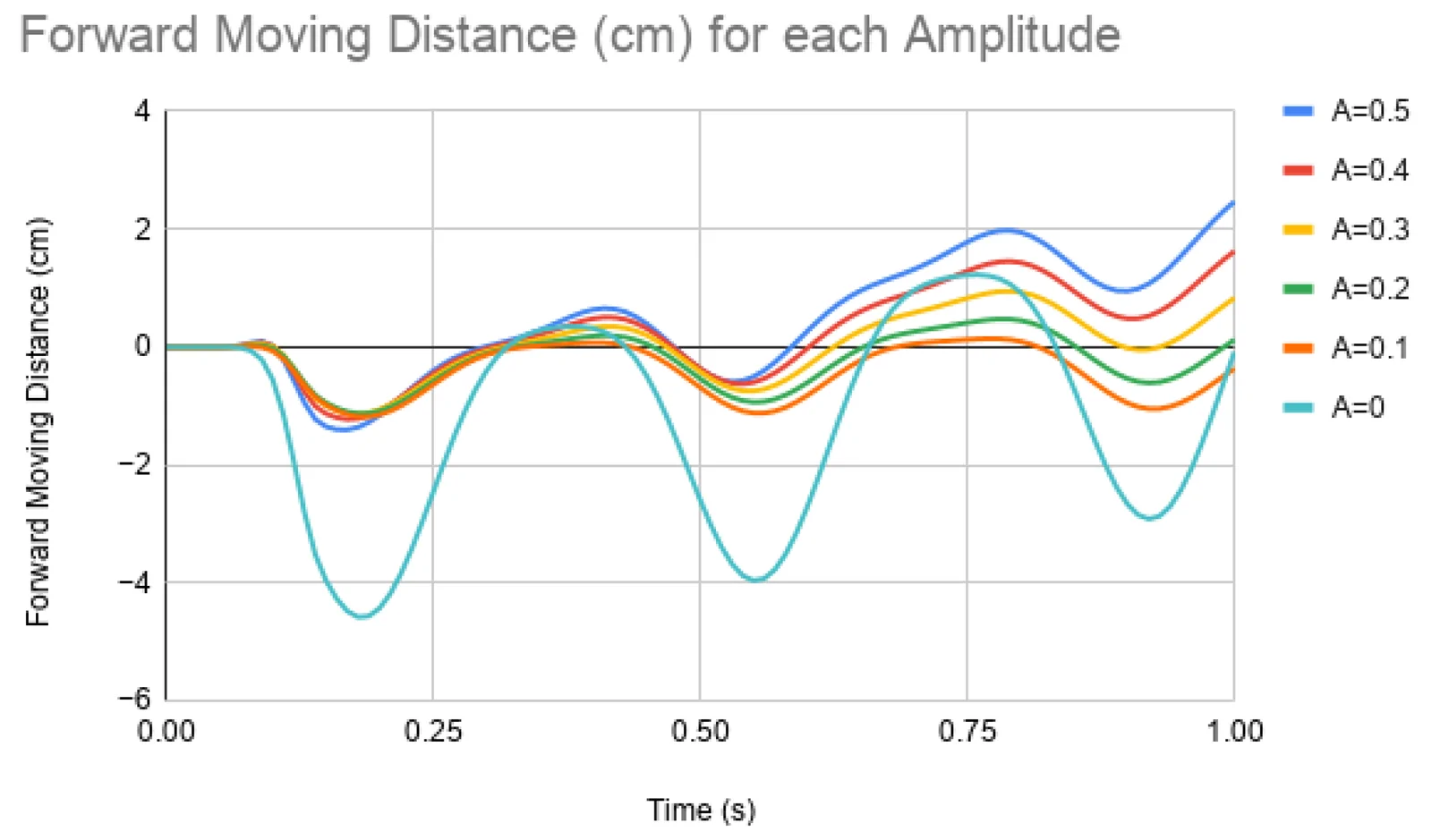
Forward moving distance (cm) over time for each amplitude of foot movement.

**Figure 9:**
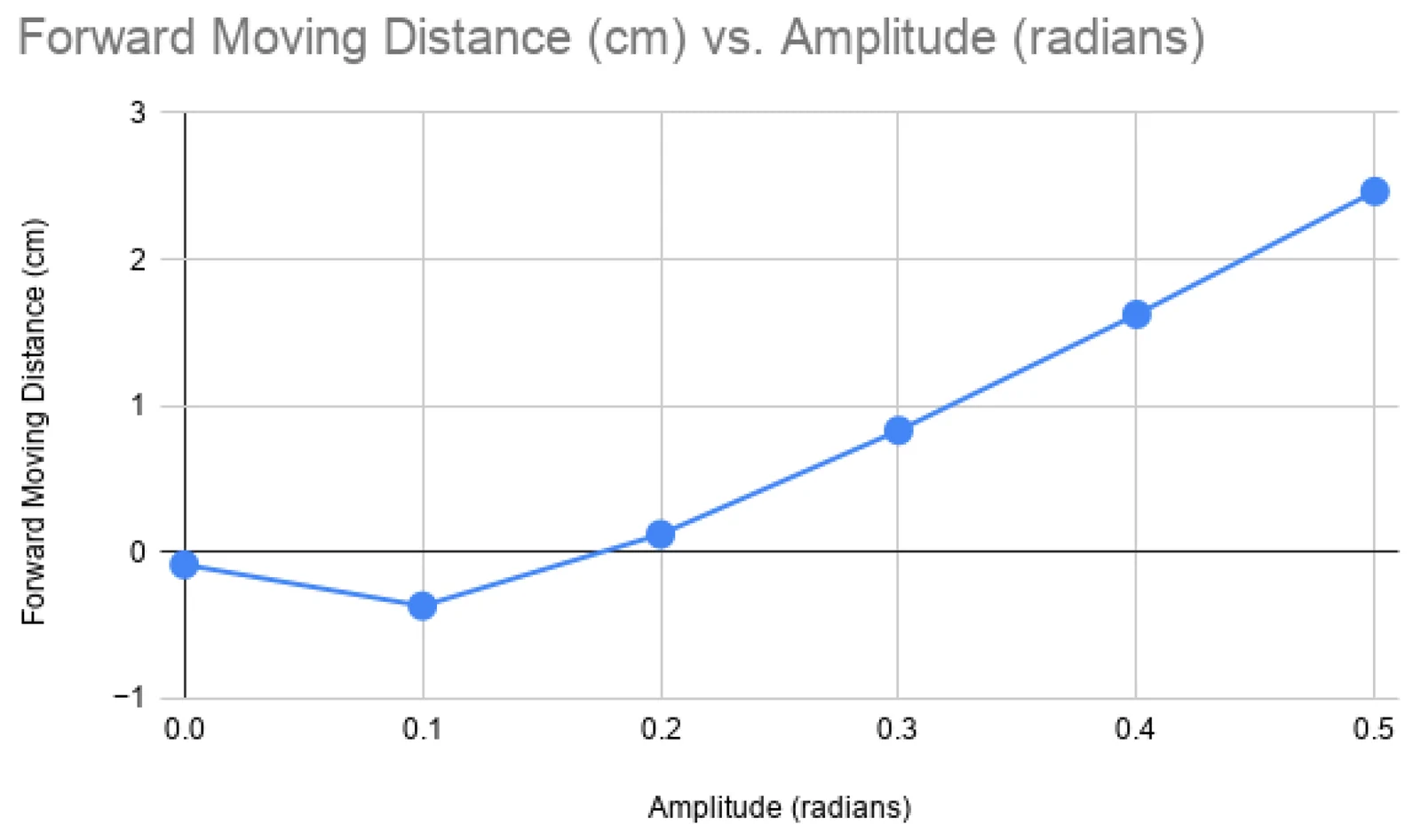
Forward moving distance (cm) at 1 s versus amplitude of foot movement.

Figure 8 shows the forward moving distance over time with varying amplitudes. The foot’s movement in a swimmer’s flutter kick significantly affects propulsion. As the foot’s movement increases with larger amplitudes, so does the propulsion, as the foot can push the water back more effectively compared to up and down movements. Although the distance values between 0 and 1 s vary with different amplitude shifts between negative and positive, there is a noticeable trend of increase. At the end of 1 s, the model with an amplitude of 0.5 radians moves 2.549 cm farther than the model with an amplitude of 0 radians, as seen in Figure 9. The distance between each amplitude increases as the amplitude gets larger. For instance, the difference in distance traveled between amplitudes of 0.4 and 0.5 radians is 0.84 cm, compared to 0.489 cm between 0.1 and 0.2 radians.

**Figure 10:**
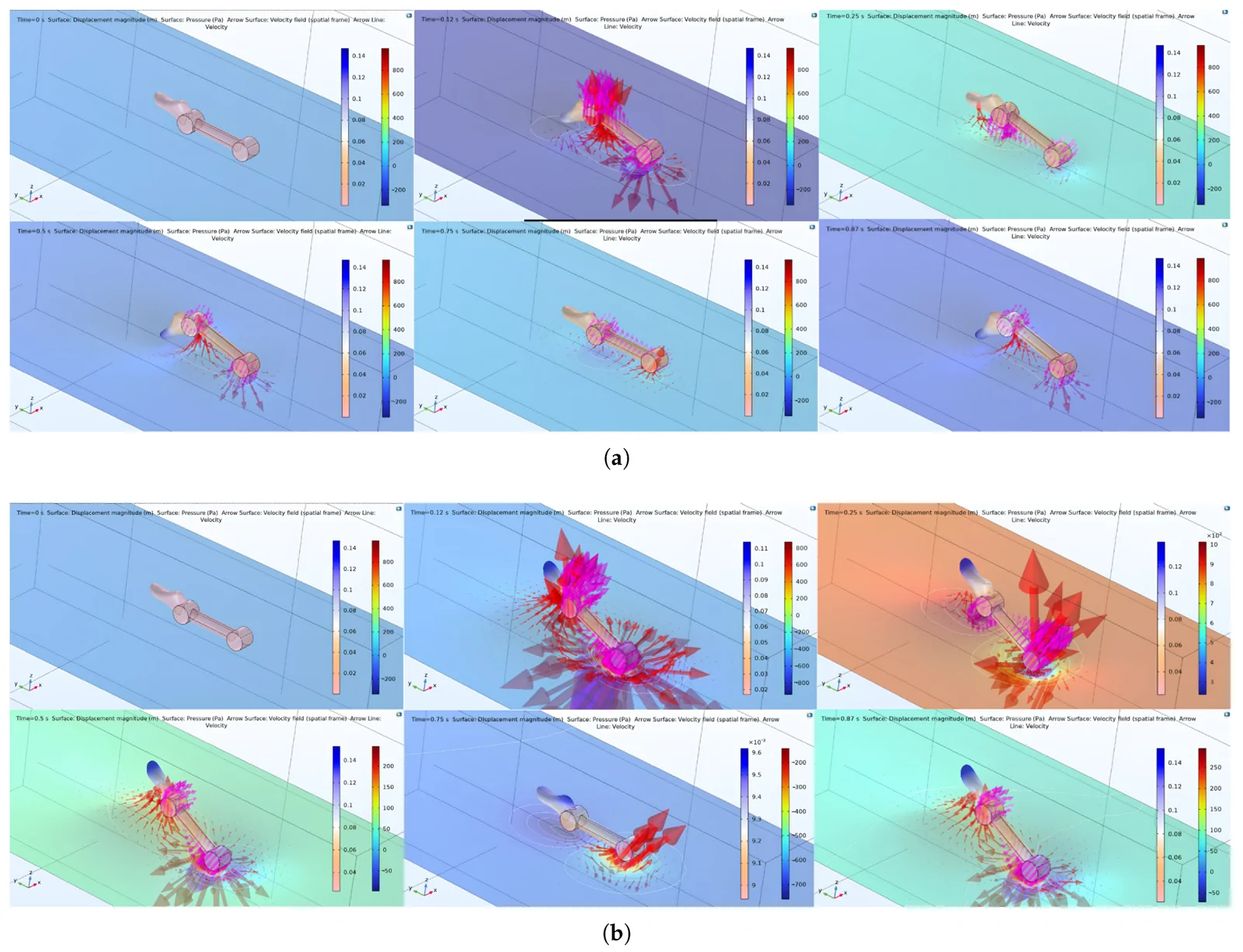
Flow diagrams of the foot and calf motion at different times. (**a**) Foot movement with an amplitude of 0.5 radians. (**b**) Foot movement with an amplitude of 0 radians.

Figure 10a,b illustrate the flow diagrams of the foot and calf motion at different times for models with amplitudes of 0.5 and 0 radians, respectively. The motion between the calf and foot is asynchronous for the model with an amplitude of 0.5 radians, while it is synchronous for the model with an amplitude of 0 radians. At 0.12 s and 0.75 s, the model with an amplitude of 0 radians experiences a significantly stronger backward force. This result aligns with participant testing trials, where a smaller spring constant in the ankle yields the highest average swimming speed. Conversely, the trial with a larger spring constant shows a lower average swimming speed, and a fixed foot performs the worst.

Therefore, proper foot movement improves both the speed and efficiency of the flutter kick.

### 3.2. Phase Difference of Foot Movement

**Figure 11:**
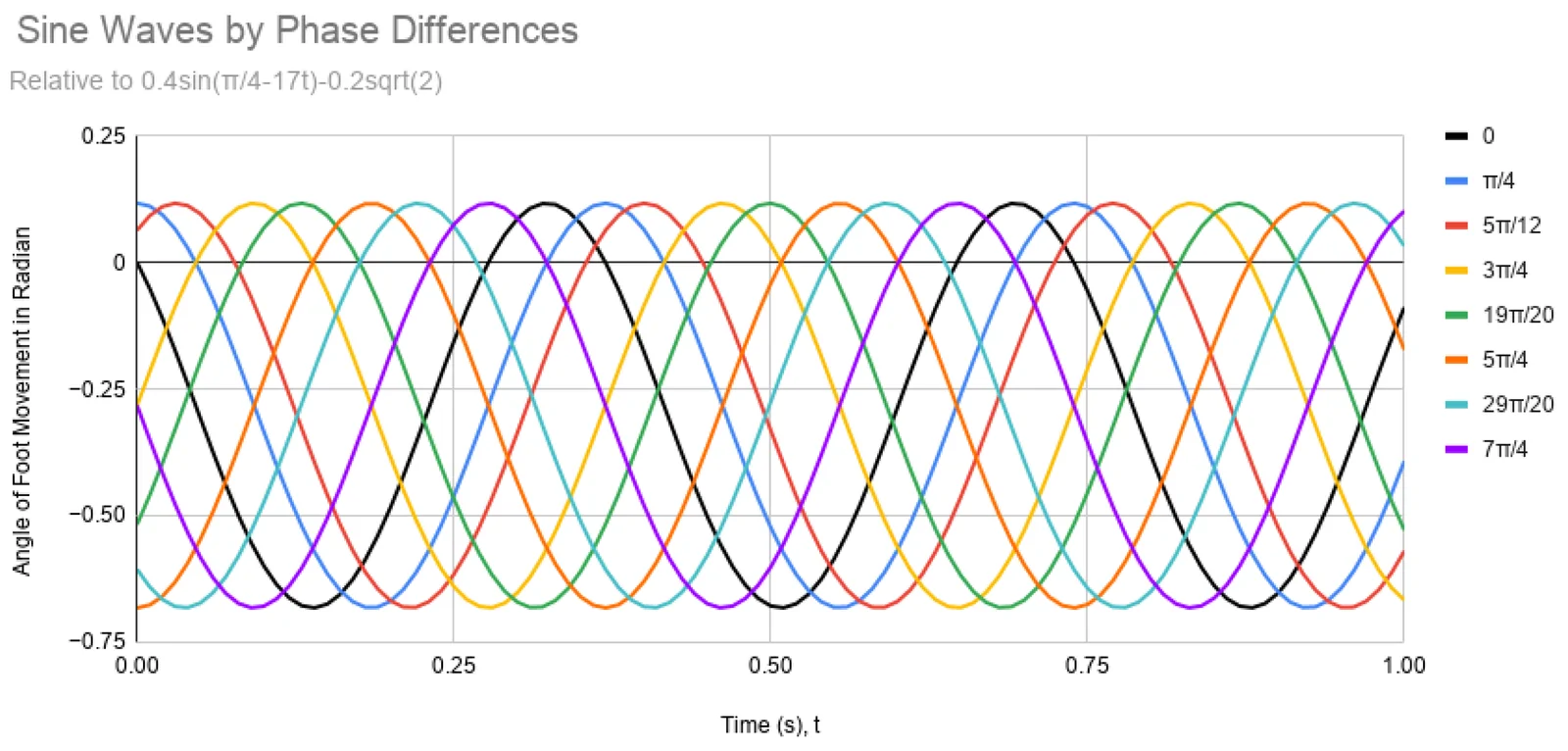
Sine waves by phase differences.

The effect of foot movement with varying phase differences relative to the original sine wave model defined in Figure 3a is assessed for phase differences of 0, π/4, 5π/12, 3π/4, 19π/20, 5π/4, 29π/20, and 7π/4, as shown in Figure 11.

**Figure 12:**
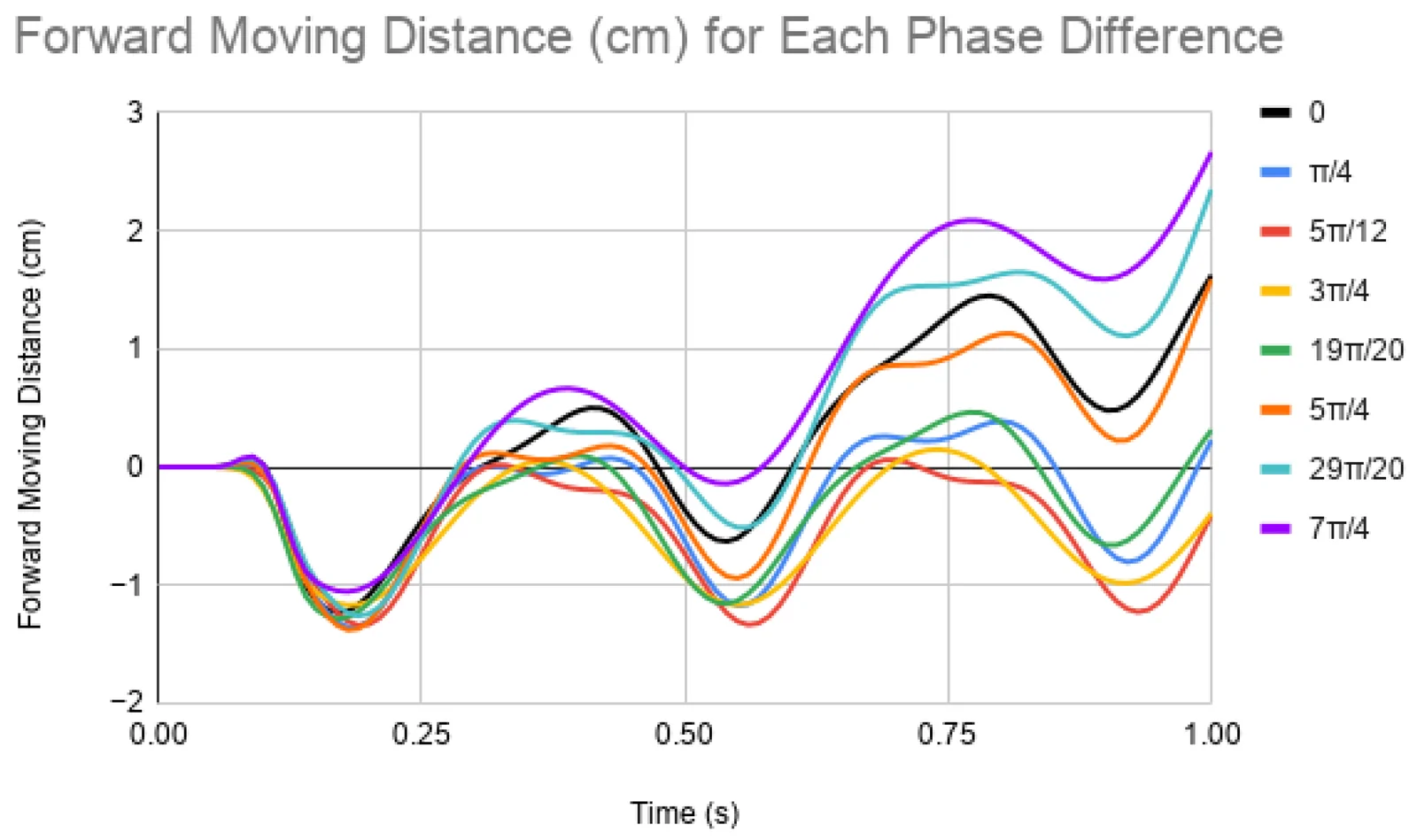
Forward moving distance (cm) over time for each phase difference of foot movement.

**Figure 13:**
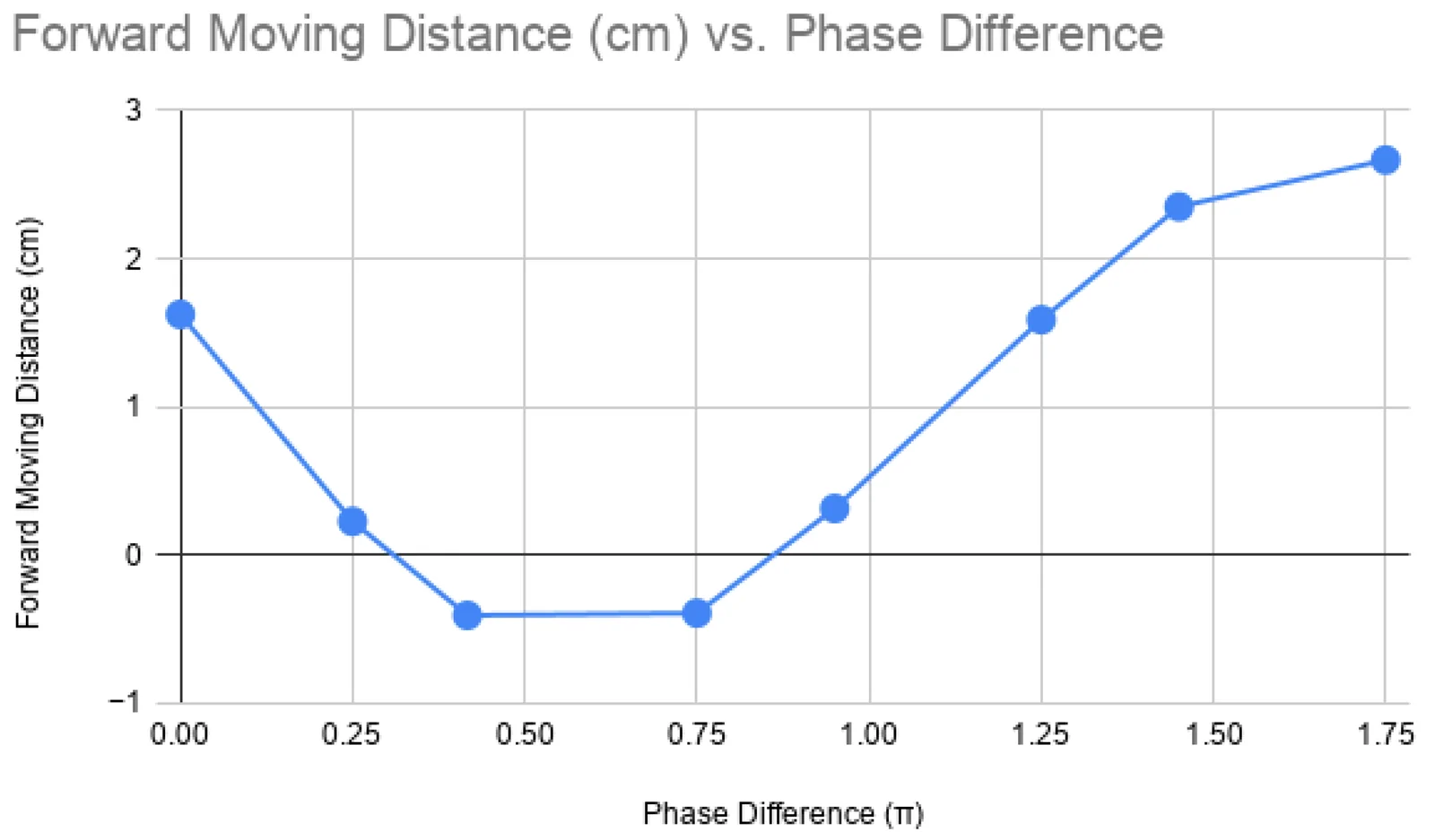
Forward moving distance (cm) at 1 s versus phase difference of foot movement.

Figure 12 illustrates the forward moving distance over time with varying phase differences. When the phase difference is 0 or greater than π, a noticeable trend of increase is observed. At the end of 1 s, the forward moving distance peaks at a phase difference of 7π/4 (2.666 cm), which is 64% greater than the original model (1.624 cm). Overall, the forward moving distance at 1 s over phase difference resembles a sine wave, as seen in Figure 13. The forward movement initially decreases from the original model with a 0 phase difference, then increases again around a phase difference of 3π/4. It gradually plateaus near 1.75π, although after 2π, the graph repeats itself, as the equation with a phase difference of 2π resembles the same graph as a phase difference of 0. A phase difference of around 0.75π results in a negative distance, as the foot exerts more force in the backward direction. Therefore, having the foot more pointed during the downward motion of the calf and flexed during the upward motion is more favorable than having the foot flexed during the downward motion and pointed during the upward motion.

**Figure 14:**
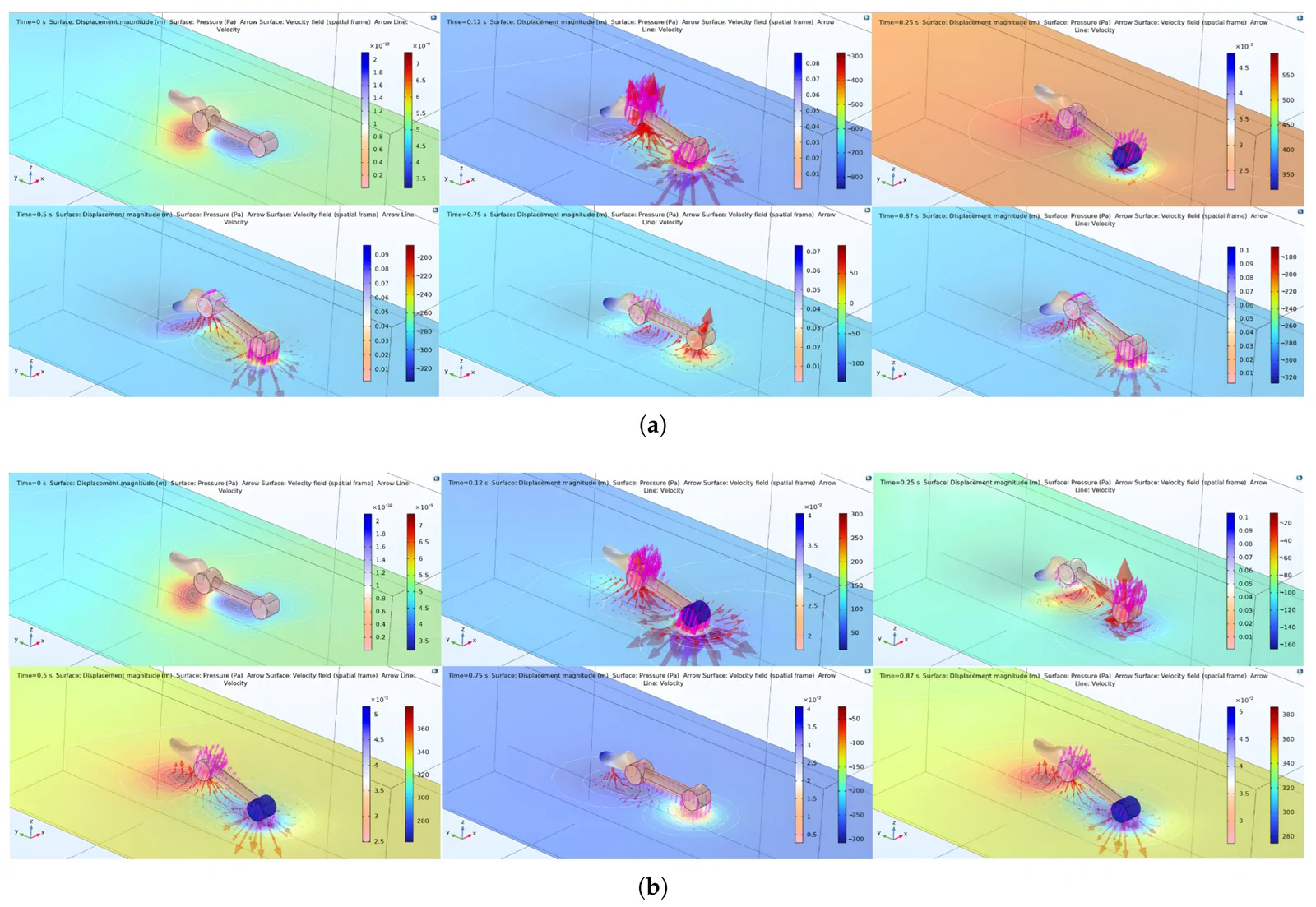
Flow diagrams of the foot and calf motion at different times. (**a**) Foot movement with a 7π/4 phase difference. (**b**) Foot movement with a 3π/4 phase difference.

Figure 14a,b illustrate the flow diagrams of the foot and calf motion at different times for foot movements with phase differences of 1.75π and 0.75π, respectively. The forward moving distance traveled in 1 s is greatest for the model with a phase difference of 1.75π, while it is least for the model with a phase difference of 0.75π. At 0.25 s, as the foot moves downwards, the model with a phase difference of 0.75π shows a greater backward force. This also applies to the upward force, which can be seen in the flow diagrams at 0.12 s.

### 3.3. Phase Difference of Foot Movement Under a Nonzero Initial Flow Velocity

The phase-difference sweep described in Section 3.2 was repeated under a nonzero initial flow condition to assess whether the optimal phase difference identified at rest also holds for a swimmer already in forward motion. For this comparison, the water velocity described in Section 2.3.2 was set to 1 m/s, directed in the same direction as the swimmer’s forward motion, and held constant throughout the 1 s simulation, while all other model parameters (amplitude of 0.4 rad, geometry, mesh, and solver settings) were unchanged from Section 3.2. This same set of phase differences (0, π/4, 5π/12, 3π/4, 19π/20, 5π/4, 29π/20, and 7π/4) was tested.

**Figure 15:**
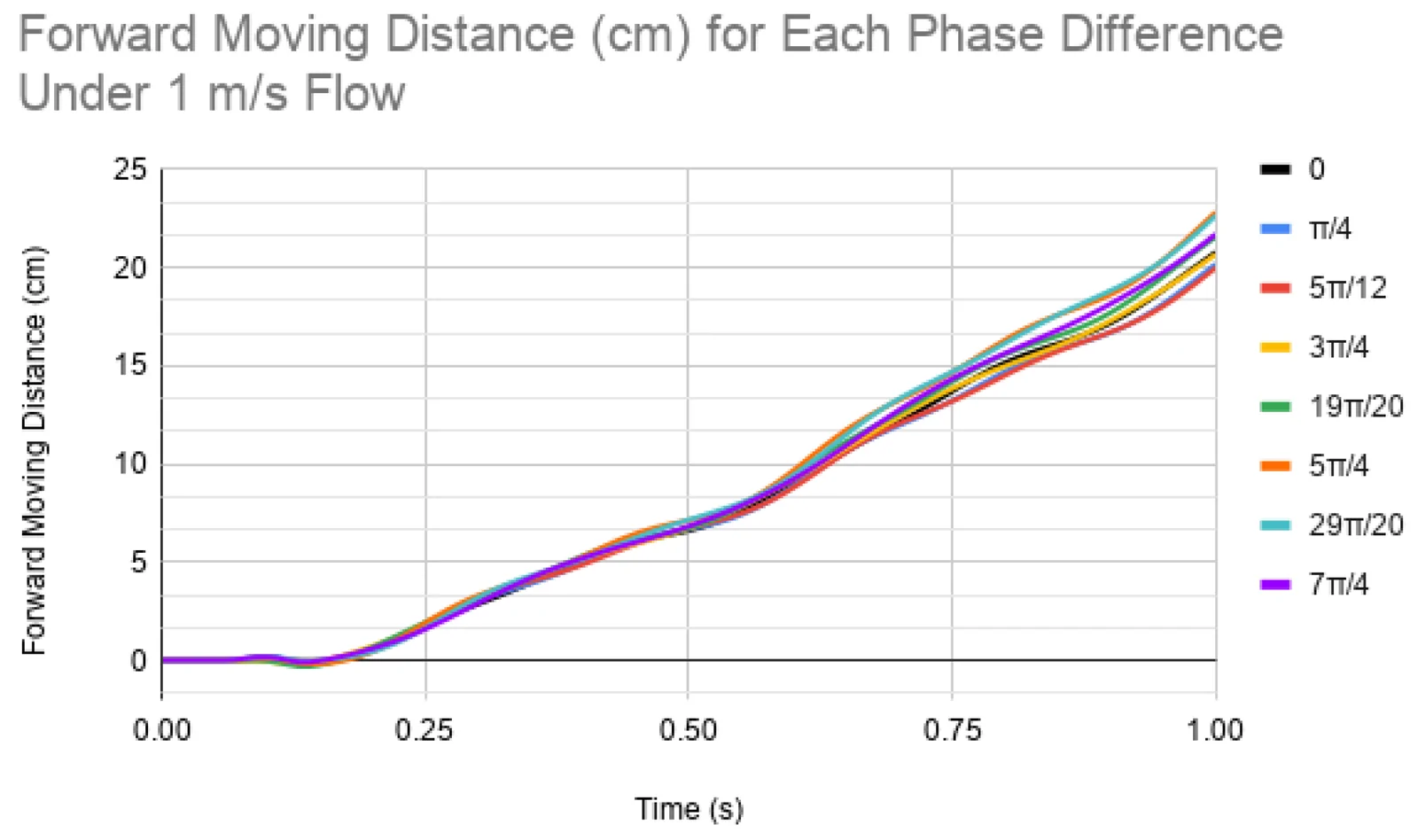
Forward moving distance (cm) for each phase difference of foot movement under a 1 m/s initial flow velocity, over the full 1 s simulation.

**Figure 16:**
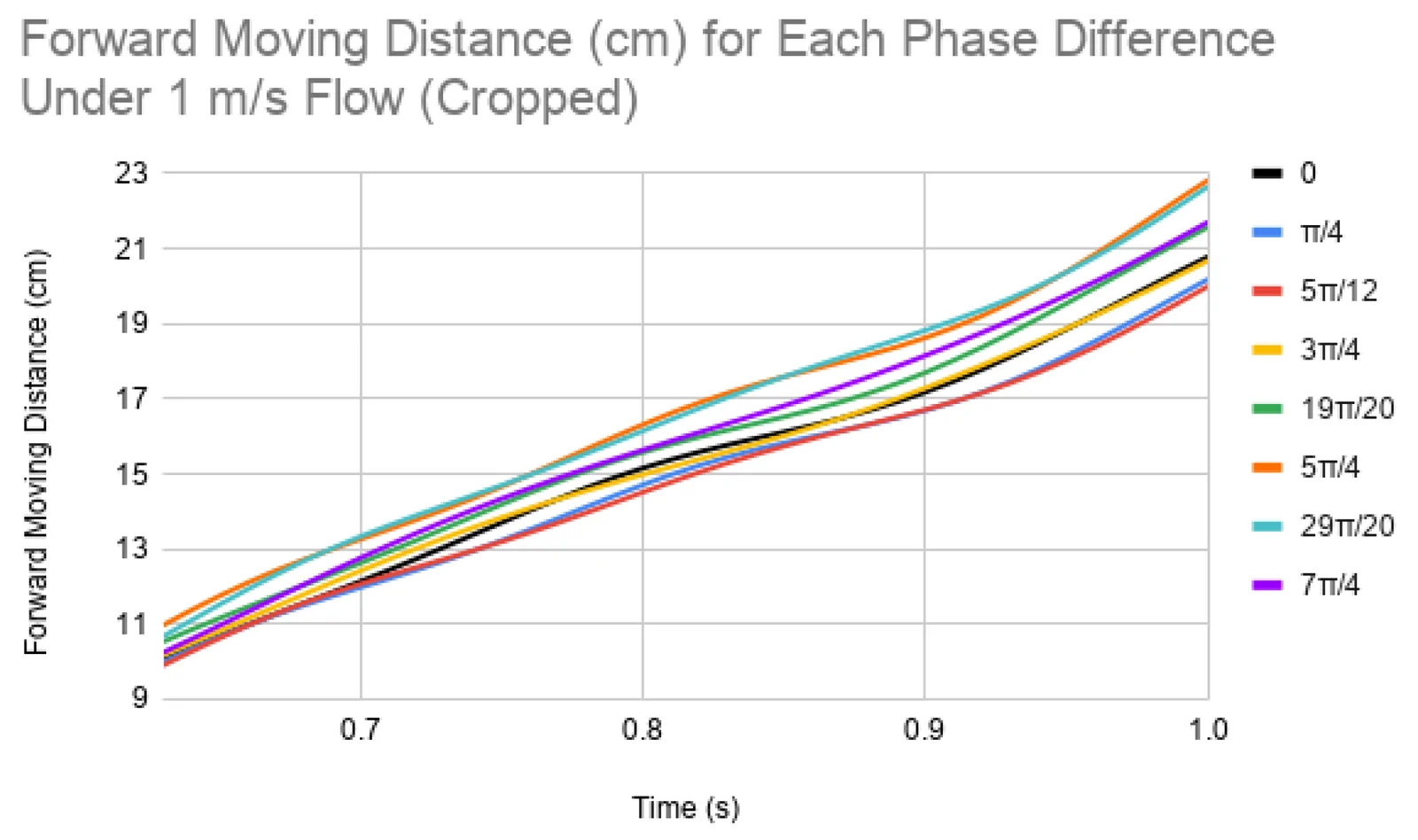
Forward moving distance (cm) for each phase difference of foot movement under a 1 m/s initial flow velocity, cropped to 0.6–1.0 s to more clearly show the relative ranking of trials near the end of the simulation. The phase difference of 5π/4 produces the greatest forward distance, followed closely by 29π/20.

Figure 15 shows the forward moving distance over time for each phase difference under this nonzero initial flow. Figure 16 shows the same data cropped to the final third of the simulation (0.6–1.0 s) to more clearly distinguish the relative ranking of the trials near the end of the simulated window.

Under the 1 m/s initial flow condition, the phase difference of 5π/4 produces the greatest forward moving distance at 1 s (22.822 cm), with 29π/20 close behind (22.65 cm), a difference of only 0.172 cm (0.75%) between the top two conditions. Notably, 7π/4, which produced the greatest forward distance under the zero-velocity initial condition in Section 3.2, falls to third place under this nonzero-flow condition, reaching 21.704 cm, 4.90% less than the new optimum of 5π/4 and 4.18% less than 29π/20. This reordering indicates that the optimal timing of foot rotation relative to the calf’s motion is sensitive to the presence of a background flow, and is not a fixed property of the kick kinematics alone. A plausible explanation is that the background flow alters the relative velocity between the foot and the surrounding fluid throughout the kick cycle, shifting the portion of the cycle in which the foot’s angle of attack generates net forward thrust rather than drag. This suggests that the ideal ankle phase timing for a swim-specific prosthetic may depend on the swimmer’s forward speed, rather than being uniformly optimal across all swimming conditions.

Therefore, our results demonstrate the substantial impact of phase difference on the distance traveled.

## 4. Discussion

Many current prosthetic designs on the market, which allow for both walking and swimming, keep the foot fixed in a flexed position or can be adjusted to remain fixed in a pointed position. Within the simplified model tested in this paper, this fixed-foot configuration was not optimal for propulsion during the flutter kick compared to configurations with a flexible, actively rotating ankle. This study demonstrates that the amplitude and phase difference of foot movement both have meaningful effects on the forward distance traveled in this idealized simulation. The technique of the sound limb, as represented by Olympian Daniel Smith, more closely resembles the higher-propulsion configurations identified here than a fixed-foot prosthetic does, suggesting that a flexible ankle capable of active rotation may better approximate the movement of the intact limb. These propulsion findings motivate, rather than establish, specific prosthetic design recommendations. They also demonstrate how matching the propulsive characteristics of the sound limb is a plausible strategy for reducing the asymmetry-related strain and overuse injuries described in the amputee musculoskeletal literature [1,2,3], though this study did not directly evaluate injury outcomes.

## 5. Limitations

This study has several limitations that should be considered when interpreting the results.

First, the geometry used in the CFD model is a simplification of the human anatomy. The calf is represented as a cylinder rather than an anatomically detailed structure (see Section 2.3.1), and although the foot geometry is retained directly from the 3D scan, the model does not resolve fine anatomical structures. These simplifications may affect fine local flow details, although the overall kick kinematics are expected to be preserved.

Second, this study exclusively models foot rotation in the sagittal plane, the up-and-down motion characteristic of the flutter kick, and does not incorporate lateral (side-to-side) rotation of the foot, which likely contributes to propulsion in healthy swimmers, even if its effect is considered secondary. The findings may not directly generalize to the different kicking kinematics of breaststroke, butterfly, or backstroke.

Third, this study reports forward knee displacement, rather than directly computed hydrodynamic thrust, as its primary propulsion metric. Displacement is monotonically related to the underlying propulsive force for a given mass and drag configuration, and is well suited to the relative comparisons made across the many trials in this study, but it is not itself a force, and it does not capture when in the kick cycle thrust is generated versus when drag dominates.

Fourth, this study uses a laminar-flow assumption rather than a turbulence model. Although we justify this assumption based on the Reynolds number and by precedent in the biological-swimming CFD literature (Section 2.3.2), the oscillating, separating motion of the kick may locally promote some transition toward turbulence that our laminar treatment does not capture.

Fifth, this study lacks direct experimental validation against independently measured propulsive forces for the specific model examined here.

## 6. Conclusions

In this study, we assess the impact of the amplitude and phase difference of foot movement in the flutter kick on the forward distance traveled. The difference in distance traveled between an amplitude of 0.5 radians and 0 radians is 2.549 cm after 1 s. For a longer swim, this distance will become much more apparent. The original movement is near the phase difference that was most favorable among the conditions tested, but the greatest forward distance among the tested phase differences and amplitudes is a 7π/4 phase shift from it, which results in a distance 64% greater at 1 s. Ultimately, as time passes, the differences in distance between different variables will grow. It should be noted that this study exclusively models foot rotation in the sagittal plane, the up-and-down motion characteristic of the flutter kick, using a simplified two-segment geometry rather than a full prosthetic device tested under real swimming conditions. Future work should consider whether incorporating multi-axis ankle movement into prosthetic design could better replicate the full range of a sound limb’s propulsive contribution. These results contribute to a better understanding of how ankle amplitude and timing influence propulsion in the flutter kick and offer a motivating rationale for developing swim-specific prosthetic ankles that more closely match the propulsion of a sound limb. Combined with the existing literature on musculoskeletal strain associated with movement asymmetry in amputees, this may help inform future prosthetic development aimed at reducing injury risk in consistent swimmers with limb loss, though injury outcomes were not directly assessed in this study.

## 7. Future Work

Several directions could extend this work. On the modeling side, future prosthetic designs and subsequent CFD studies should consider whether accounting for lateral rotation, multi-axis ankle movement, and other competitive strokes changes the optimal ankle mechanics identified here. Incorporating a turbulence model (e.g., the k-ω model) would allow the sensitivity of the propulsion trends to the laminar-flow assumption to be tested directly, and reporting hydrodynamic thrust directly, rather than forward knee displacement, is a natural extension of this analysis.

On the validation side, direct experimental validation against independently measured propulsive forces represents an important next step. Relatedly, extending the transient analysis presented here, reporting force output directly rather than displacement over longer simulated durations, and jointly varying phase, amplitude, and forward speed would parallel established practice in the CFD literature on multi-finned swimming robots.

## Figures and Tables

**Table 1:** Experimental test conditions used in the CFD simulations.

Trial	Sweep	Amplitude (rad)	Phase Difference
1	Amp.	0	0 (baseline) *
2	Amp.	0.1	0 (baseline) *
3	Amp.	0.2	0 (baseline) *
4	Amp.	0.3	0 (baseline) *
5	Amp./Phase	0.4 (original)	0 (baseline) *
6	Amp.	0.5	0 (baseline) *
7	Phase	0.4 (original)	π/4
8	Phase	0.4 (original)	5π/12
9	Phase	0.4 (original)	3π/4
10	Phase	0.4 (original)	19π/20
11	Phase	0.4 (original)	5π/4
12	Phase	0.4 (original)	29π/20
13	Phase	0.4 (original)	7π/4

* “0 (baseline)” indicates the original, video-fitted kicking motion (Figure 3a), which is not itself a swept phase difference.

## Data Availability

The original contributions presented in the study are included in the article; further inquiries can be directed to the corresponding author.

## Abbreviations

The following abbreviations are used in this manuscript:

CFD	Computational Fluid Dynamics
N-S	Navier–Stokes
